# Case Report: Fecal microbiota transplantation via capsules ameliorated clinical outcomes in a patient with multiple sclerosis

**DOI:** 10.3389/fimmu.2025.1678759

**Published:** 2025-11-26

**Authors:** Stefano Bibbò, Flavio De Maio, Fioravante Capone, Gianluca Quaranta, Debora Rondinella, Roberto Rosato, Mauro Minelli, Desy De Lorenzis, Maurizio Sanguinetti, Giovanni Cammarota, Vincenzo Di Lazzaro, Luca Masucci

**Affiliations:** 1 Department of Medical and Surgical Sciences, Unità Operativa Complessa (UOC) Gastroenterologia, Fondazione Policlinico Universitario Gemelli Istituto di Ricovero e Cura a Carattere Scientifico (IRCCS), Rome, Italy; 2 Department of Laboratory and Haematological Sciences, Fondazione Policlinico Universitario A. Gemelli Istituto di Ricovero e Cura a Carattere Scientifico (IRCCS), Rome, Italy; 3 Department of Medicine and Surgery, Unit of Neurology, Neurophysiology, Neurobiology and Psychiatry, Università Campus Bio-Medico di Roma, Roma, Italy; 4 Department of Legal and Business Sciences. Università Libera Università Mediterranea (LUM) Giuseppe Degennaro. Casamassima, Bari, Italy; 5 Department of Basic Biotechnological Sciences, Intensive and Perioperative Clinics, Università Cattolica del Sacro Cuore, Rome, Italy; 6 Department of Translational Medicine and Surgery, Università Cattolica del Sacro Cuore, Rome, Italy; 7 Fondazione Policlinico Universitario Campus Bio-Medico, Roma, Italy

**Keywords:** case report, multiple sclerosis, fecal microbiota transplantation, autoimmunity, bacterial therapy

## Abstract

Multiple Sclerosis (MS) has long been recognized as a multifactorial disease, associated with both genetic and enviromental factors. Its link to inflammatory processes has led to significant advances in understanding the immunological and neurobiological mechanisms underlying the disease. The presumed autoimmune etiology is currently guiding the development of therapeutic protocols in this direction. The intestinal bacteria population, known as Gut Microbiota (GM), plays a well-documented role in autoimmune and inflammatory diseases. Gut microbiota dysbiosis is associated in patients affected by MS. Fecal Microbiota Transplantation (FMT) is emerging as a potential strategy to restore eubiosis and modulate systemic inflammation. We treated a 42-year-old woman with severe MS disability by FMT via colonoscopic infusion followed by a 3-month treatment of daily oral capsules, containing frozen microbiota, in order to resolve gastrointestinal symptoms. Clinical follow-up was conducted at 30, 60, and 90 days. Microbiota profiling (16S rRNA sequencing) and intestinal permeability testing were performed at several time points before and post infusion. Post-FMT, gastrointestinal symptoms improved significantly, as well as in limb spasticity, trunk stability, and fine motor skills. Microbiota analysis revealed a marked reduction in the abundance of *Akkermansia muciniphila* (22.5% vs 6.6%). At phylum level, Actinomycetota remained elevated (22%), while Bacteroidota consistently showed low abundance (14%). The most favorable microbiota profile was observed at 90 days, which coincided with the peak of clinical improvement. Intestinal permeability also improved over time, despite the patient’s microbiota profile remaining distinct from the donor. This is the first report about combined FMT in MS. The procedure was safe, well tolerated, and associated with both gastrointestinal and neurological improvements. These findings support further exploration of FMT as a therapeutic adjunct in MS through controlled clinical trials.

## Introduction

Multiple sclerosis (MS) is a disease of the central nervous system (CNS), also associated to autoimmune dysregulation. For this reason, recent therapeutic protocols include treatments targeting the underlying mechanisms (1). The intestinal bacterial population, gut microbiota (GM), plays a key role in immunomodulation (2). The GM profile is altered in MS (3). In recent years, the fecal microbiota transplantation (FMT) has proven to be a valid therapeutic option for the restoring of intestinal eubiosis in *Clostridioides difficile* (4) infection and in autoimmune diseases (2). MS patients also report intestinal discomfort which may be due to both alterations in bacterial composition and neurological involvement (5).

Basing on our findings, the efficacy of FMT in patients with MS has documented in only a few cases (6). We report the first case of a patient affected by MS treated using a combined FMT approach via colonoscopy and oral capsules, aimed at alleviating gastrointestinal symptoms. To assess the efficacy of the procedure, next-generation sequencing (NGS) was performed on fecal samples collected before the infusion of microbiota and over a five-month period. Moreover, clinical assessments were carried out pre-FMT and at monthly intervals for three months. This interventional approach was effective in normalizing intestinal function and improving the patient’s neurological status.

## Case

We describe a 42-year-old female affected by secondary progressive MS (SPMS). Disease onset was in 2009 with lower limbs impairment. Since 2018, she was restricted to wheelchair. The patient underwent multiple treatment protocols, including interferon β-1A, Natalizumab (Tysabri^®^), Teriflunomide (Aubagio^®^), Methotrexate, and Simvastatin (for its potential neuroprotective effects), all of which were discontinued due to lack of clinical efficacy or poor tolerability. She is currently not receiving any immunosuppressive therapy and has not been under such treatment for approximately 10 years; furthermore, she did not resume any immunosuppressive therapy after the infusion procedure.

The most recent brain MRI showed stable multiple demyelinating lesions without Gadolinium-enhancement.

From an immuno-allergological perspective, the patient had reduced MTHFR enzymatic activity and a positive HLA-DQ7 haplotype, factors that may predispose to intestinal inflammation (7) and food intolerances (8). Documented intolerances to nickel sulfate and cobalt chloride are associated with clinical symptoms following metal exposure.

The patient reported longstanding chronic gastrointestinal symptoms, including postprandial abdominal cramping, foul-smelling flatulence, urgent bowel movements, and altered bowel habits. Additional symptoms included dyspepsia, intermittent constipation, and delayed gastric emptying.

She experienced systemic and vascular symptoms, such as cold extremities, thermal dysregulation, muscle stiffness, fatigue, slowed movements, and neurovegetative disturbances.

The patient underwent fecal microbiota transplantation (FMT) through the following protocol: an initial infusion was administered via colonoscopy using a 200 mL solution derived from a healthy donor. Starting the day after the infusion, the patient began oral administration of frozen capsules containing microbiota from the same donor, at a dosage of three capsules per day (10^6^–10^9^ CFU/mL) for a duration of three months. (**Table 1**). No antibiotic or probiotic therapy was administered to the patient in the five months prior to the procedure.

**Table 1:** Clinical follow-up and FMT administration.

Time point	Functional/clinical notes	FMT administration
Pre-FMT Evaluation	Severe impairment, limited passive mobility; high disability	Initial infusion via colonoscopy (200 mL donor microbiota solution); start of oral capsules 3/day (10^6–10^9 CFU/mL) from day after infusion
30-Day Follow-up Post-FMT	Initial clinical improvement; standing more stable; physiotherapy ongoing	Continuation of oral capsules 3/day
60-Day Follow-up Post-FMT	Improved autonomy in fine motor tasks; independence in some daily activities	Continuation of oral capsules 3/day
90-Day Follow-up Post-FMT	Improved GI symptoms, thermoregulation, and food tolerance; physiotherapy mostly active	End of oral capsules course (3 months)

### Donor selection

The donor selection phase for fecal microbiota transplantation (FMT) consists of a multi-step screening process (9). Donors are initially evaluated through an anamnesis-based questionnaire aimed at excluding risk factors for transmissible diseases and relevant comorbidities, including neurological diseases. Subsequently, the potential donor undergoes a rigorous series of serological and microbiological screening tests performed on blood and stool samples.

Blood and serum analyses include testing for Cytomegalovirus (CMV), Epstein–Barr virus (EBV), hepatitis viruses A (HAV), B (HBV), C (HCV), and E (HEV), HIV-1/2, and syphilis (*Treponema pallidum*), as well as general blood chemistry tests (complete blood count, C-reactive protein [CRP], erythrocyte sedimentation rate [ESR], albumin, creatinine, electrolytes, aminotransferases [ALT, AST], bilirubin, gamma-glutamyl transferase [GGT], and alkaline phosphatase).

In parallel, stool samples are examined using cultural, molecular, and microscopic methods to exclude intestinal pathogens such as bacteria (Salmonella spp., Campylobacter spp., Shigella spp., Yersinia spp., *Escherichia coli* O157:H7), viruses, protozoa, and helminths, as well as *Clostridioides difficile* toxin A/B-producing strains, detected by chemiluminescence assays. Additional tests are also performed to assess fecal occult blood, calprotectin, and *Helicobacter pylori* fecal antigen.

Furthermore, screening includes the evaluation of possible colonization by multidrug-resistant organisms (MDROs), such as vancomycin-resistant *Enterococcus faecium* and *Enterococcus faecalis* (VRE), methicillin-resistant *Staphylococcus aureus* (MRSA), and Gram-negative bacteria producing carbapenemases or beta-lactamases, as well as *Listeria monocytogenes*. Starting from March 2020, due to the COVID-19 pandemic, the screening protocol was updated to include the detection of SARS-CoV-2 in fecal samples.

### FMT solution

Two aliquots of 50 g of fecal material were prepared. Each aliquot was suspended in 150 mL of 0.9% NaCl sterile saline solution supplemented with 10% glycerol ((MONICO s.p.a.- Venezia). The subsequent filtration step was carried out using sterile plastic bags with an integrated gauze filter, STOMACHER^®^ 400 Circulator Strainer Bags (Seward, UK), and homogenized with a STOMACHER^®^ 400 Circulator (Fisher Scientific, Waltham, USA) at a speed of 230 rpm for 30 seconds (10).

The suspension resulting from the filtration of each aliquot was transferred into dedicated 200 mL sterile containers. One aliquot was stored at –80 °C, while the other was used for the preparation of frozen capsules. In addition, two further aliquots (1.5 ml) were stored for possible quality control testing.

### Capsule preparation

Gastro-resistant capsules, size <ns/>1 (FARMALABOR SRL – Canosa di Puglia, BT), were filled with 400 µL of fecal suspension. Than each size <ns/>1 capsule was inserted into a gastro-resistant capsule, size <ns/>00 (FARMALABOR SRL – Canosa di Puglia, BT). The prepared capsules were stored in a freezer at −80 °C (11).

### Pre-FMT evaluation

The patient presented with a severe neurological condition, characterized by marked spastic hypertonia of the lower limbs and the left upper limb. Ambulation was absent, while upright posture was possible only for a few seconds with bilateral support. Neurological examination revealed bilateral Babinski sign and deep tendon reflexes hyperactive in the lower limbs and hypoactive in the upper limbs. Motor function in the right upper limb was partially preserved, whereas the left upper limb was severely impaired. Spasticity was evident at lower limbs and left arm, limiting passive mobilization of arms. The Expanded Disability Status Scale (EDSS) score was 8.

### 30-day follow-up post-FMT

The patient reported initial clinical improvement, including mild reduction in spasticity affecting limbs and increased stability while standing. Neurological examination confirmed the improved ability to maintain an upright posture with bilateral support (although ambulation remained impossible), the presence of brisk reflexes in the lower limbs, and no deterioration in sensory function or emergence of new neurological deficits.

### 60-day follow-up post FMT

The patient regained fine motor skills in the right hand, now able to hold a pen and sign her name. She is also capable of combing her hair and washing her face independently. All other neurological findings remain unchanged.

### 90-day follow-up post-FMT

The most recent evaluation documented further clinical improvement. The patient was able to maintain upright posture with greater stability compared to previous assessments and reported a more marked reduction in spasticity, particularly in the lower limbs. Both subjective and objective benefits were observed from physiotherapy, which is now conducted primarily in active mode. The patient reported improved food tolerance, gastrointestinal symptoms and thermoregulation. A summary is reported in **Table 1**.

**Figure 1:**
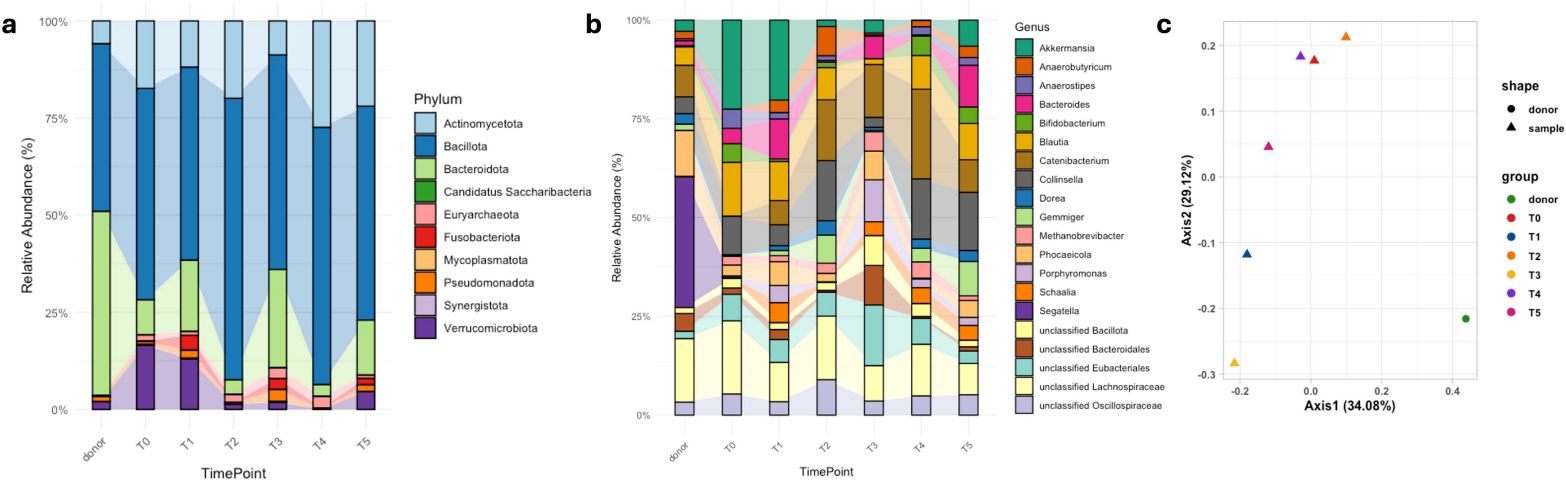
Microbiota profiling of the patient’s samples before (T0) and following Faecal Material Transplantation every 30 days (T1, T2, T3, T4, T5) and donor’s sample characterization. **(a)** shows relative abundances at phylum level. **(b)** shows relative abundances of the top 20 genera, while **(c)** represents Bray Curtis beta diversity represented as Principal Coordinate analysis.

To assess the modulation of the gut microbiota, six stool samples from the patient and the donor sample were analyzed using 16s metabarcoding and analyzed as described above (12). The samples were collected prior to the colonoscopic infusion (T0) and then monthly for five consecutive months. At baseline (T0), the microbiota profile showed a marked increase in Verrucomicrobiota (17%) and Actinomycetota (17%), along with a reduced abundance of Bacteroidota (9%) and an elevated proportion of Bacillota (54%). Concerning these, two peakes in the latter were observed at T2 (73%) and T4 (66%), followed by a return to baseline level. This variation coincided with a marked decrease in Bacteroidota, with relative abundances of 4% and 3% respectively (**Figure 1a**).At the genus level, a notably high abundance of *Akkermansia* (23%) was observed at T0; however, its levels remained consistently reduced and closer to normal values in all post-FMT time points. Among other genera, *Collinsella* showed a relative abundance of 9.8% at T0, which dropped to 2.5% at T3, and subsequently rose to around 15% in the following time points. *Blautia* exhibited fluctuating values between 8% and 14%, except at T3, where it showed a marked decrease to 1.4%. Additionally, *Phocaeicola* had a low relative abundance at T0 (2.8%), peaked at T3 (7.2%), dropped again at T4 (1.7%), and then stabilized at 4.3% at T5. Unclassified Bacteroidales and Unclassified Eubacteriales were more represented always in T3 (10% and 15.3%, starting from 1.5% and 6% respectively), while Unclassified Lachnospiraceae decreased (9% vs 18.5% at T0) (**Figure 1b**). Interestingly, Bray Curtis beta diversity highlighted that the patient’s samples remained consistently distant from the donor’s profile (**Figure 1c**). However, intestinal permeability, assessed by the lactulose-mannitol test, showed a mannitol value at before FMT (193.0 mg) higher than at four months (<0.2 mg)) by HPLC PAD method.

## Discussion

Studies evaluating the efficacy and safety of FMT capsules primarily relate to their use in *Clostridioides difficile* infections. In a meta-analysis including a total of 664 patients, most adverse events were mild gastrointestinal symptoms such as nausea, diarrhea, and abdominal pain. Only 14 serious adverse events were reported, all of which were attributed to pre-existing comorbidities, except for two cases of hospitalization due to recurrent *Clostridioides difficile* infection. Therefore, no serious adverse events were considered potentially attributable to oral FMT capsules (13). With regard to patients affected by multiple sclerosis, we can only hypothesize that their use should first be evaluated by a neurologist to rule out any potential issues related to swallowing.

This report documents, to the best of our knowledge, the first combined FMT, via colonoscopy and oral capsules, in a patient with SPMS, primarily aimed to treat gastrointestinal symptoms. This approach, which combines direct enteral delivery with oral administration, allows for an initial deep colonization of the gut followed by prolonged maintenance of the donor microbiota. MS is recognized as a multifactorial disease in which immune dysregulation plays a central role. The gut microbiota is now well established as a key player in the immune modulation and therefore in the development of autoimmune diseases, including MS (14). Particularly, it has been clearly demonstrated that, in these patients, the abundance of butyrate-producing bacteria is reduced (15). Butyrate belongs to the group of bacterial metabolites known as short-chain fatty acids (SCFAs), which play a role in the differentiation of regulatory T cells (Tregs) in the gut district (16). Recently, a study documented that butyrate induces the expansion of CD69^+^CD56^dim^ NK cells and enhances the cytotoxicity of CD56^bright^ NK cells against hyperactivated CD25^+^CD69^+^ T cells, which are potentially harmful in autoimmune settings, while modulating the responce toward overall autologus CD4^+^ T cell compartment (17). In this context, other studies have highlighted how specific bacterial taxa are observed in MS. *Akkermansia muciniphila* is particularly over-represented in MS patients compared to healthy controls (14). A proposed hypothesis is that, due to its specific mucin-degrading activity, an increased abundance of this strain may enhance its effect on the mucosal layer, thereby exerting a pro-inflammatory action (18). Similarly, *Collinsella* genus and other bacteria has been associated with worse disease course, also evident on radiological evaluation (19). Considering this background, FMT emerges as a promising immunomodulatory strategy even in MS patients, documented only in limited number of cases (6). In the present case, patient’s neurological condition showed a positive trend particularly in spasticity and balance, following FMT and the maintenance of physiotherapy. Spasticity was reduced, upright posture became more stable and sustained, and some subtle objective neurological parameters improved. The main clinical response was observed 90 days after beginning of procedure. The efficacy of FMT is further supported by 16S rRNA gene sequencing analysis. Examining the evaluated time points, the most favorable microbiota redistribution was observed at T3, precisely 90 days. At the phylum level, this was characterized by a decrease in Actinomycetota and Verrucomicrobiota, accompainied by an increase in Bacteroidota. This pattern reflected a reduction in *Akkermansia muciniphila* and *Collinsella* genus and a rise in *Phocaeicola* genus and unclassified Bacteroidales. The association of *Akkermansia muciniphila* and *Collinsella* with MS have been previously described. Regarding Unclassified Bacteriodales, we can only state that they contribute to raising the overall level of Bacteroidota.*Phocaeicola* reflects the update taxonomy of *Parabacteroides* genus and certain strains may confer favorable effects by the production of SCFAs (20) Unclassified Eubacteriales showed an increase, potentially contributing to beneficial effects through the production of SCFAs, including butyrate (21). The use of encapsulated microbiota was well tolerated by the patient and may contribute to maintaining a protective effect mainly against the overgrowth of *Akkermansia muciniphila* or other yet-to-be-identified species. Importantly, the fluctuations observed in microbiota profiles are likely attributable to well-known confounding factors (genetic, dietary, or environmental) which can hinder the identification of biomarkers and impair successful engraftment of the donor microbiota. Indeed, the comparison with the donor sample revealed a stable distance between microbial profiles, despite prolonged microbiota administration. However, the overall improvement suggests a beneficial effect of FMT, which can be considered an adjunctive approach when combined with intensive physiotherapy and standard care protocols.

## Conclusion

The few cases reported to date, including the present one, consistently show a shift toward a more balanced microbial profile and the potential role of FMT in modulating gut-immune-CNS axis. Moreover, the use of capsules containing intestinal microbiota represents a clear advantage in the FMT procedure, as they can be taken by the patient at home. However, the major limitation of this study is that it evaluates a single patient. This makes it impossible to identify potential microbial species-level clusters associated with MS, which can only be determined through comparison across multiple subjects by metagenomic. Therefore larger, controlled or pilot studies are required to improve the robustness of these preliminary finding.

## Data Availability

The data presented in this article have been deposited with NCBI BioProject, accession number PRJNA1365666.
