# Correction: Case Report: Fecal microbiota transplantation via capsules ameliorated clinical outcomes in a patient with multiple sclerosis

**DOI:** 10.3389/fimmu.2025.1768227

**Published:** 2026-01-02

**Authors:** Stefano Bibbò, Flavio De Maio, Fioravante Capone, Gianluca Quaranta, Debora Rondinella, Roberto Rosato, Mauro Minelli, Desy De Lorenzis, Maurizio Sanguinetti, Giovanni Cammarota, Vincenzo Di Lazzaro, Luca Masucci

**Affiliations:** 1 Department of Medical and Surgical Sciences, Unità Operativa Complessa (UOC) Gastroenterologia, Fondazione Policlinico Universitario Gemelli Istituto di Ricovero e Cura a Carattere Scientifico (IRCCS), Rome, Italy; 2 Department of Laboratory and Haematological Sciences, Fondazione Policlinico Universitario A. Gemelli Istituto di Ricovero e Cura a Carattere Scientifico (IRCCS), Rome, Italy; 3 Department of Medicine and Surgery, Unit of Neurology, Neurophysiology, Neurobiology and Psychiatry, Università Campus Bio-Medico di Roma, Roma, Italy; 4 Department of Legal and Business Sciences. Università Libera Università Mediterranea (LUM) Giuseppe Degennaro, Bari, Italy; 5 Department of Basic Biotechnological Sciences, Intensive and Perioperative Clinics, Università Cattolica del Sacro Cuore, Rome, Italy; 6 Department of Translational Medicine and Surgery, Università Cattolica del Sacro Cuore, Rome, Italy; 7 Fondazione Policlinico Universitario Campus Bio-Medico, Roma, Italy

**Keywords:** case report, multiple sclerosis, fecal microbiota transplantation, autoimmunity, bacterial therapy

Affiliation “3 Department of Medicine and Surgery, Unit of Neurology, Neurophysiology, Neurobiology and Psychiatry, Università Campus Bio-Medico di Roma, Roma, Italy” was erroneously given as “Research Unit of Neurology, Department of Medicine and Surgery, Università Campus Bio-Medico di Roma, Via Alvaro del Portillo, Roma, Italy”.

Affiliation “7 Fondazione Policlinico Universitario Campus Bio-Medico, Roma, Italy” was erroneously given as “Fondazione Policlinico Universitario Campus Bio-Medico, Via Alvaro del Portillo, Rome, Italy”.

Author “Maurizio Sanguinetti” was erroneously assigned to “affiliations 3 and 4”. These affiliations have now been removed for author “Maurizio Sanguinetti”.

Author “Giovanni Cammarota” was erroneously assigned to “affiliations 2, 3, 4 and 5”. These affiliations have now been removed for author “Giovanni Cammarota”.

Author “Vincenzo Di Lazzaro” was erroneously assigned to “affiliations 4, 5 and 6”. These affiliations have now been removed for author “Vincenzo Di Lazzaro”.

Author “Luca Masucci” was erroneously assigned to “affiliations 3 and 4”. These affiliations have now been removed for author “Luca Masucci”.

The original version of this article has been updated.

